# Overexpression of miR-375 Protects Cardiomyocyte Injury following Hypoxic-Reoxygenation Injury

**DOI:** 10.1155/2020/7164069

**Published:** 2020-01-03

**Authors:** Md Sayed Ali Sheikh

**Affiliations:** Department of Internal Medicine, Cardiology, College of Medicine, Jouf University, Sakaka, Al Jouf, Saudi Arabia

## Abstract

The aim of the study was to evaluate the clinical significance of microRNA-375 in acute myocardial infarction patients and its mimic action in hypoxia/reoxygenation- (H/R-) induced ventricular cardiomyocyte H9c2 injury. In the current study, 90 ST-elevated acute MI patients (STEMI), 75 non-ST-elevated acute MI patients (NSTEMI), 90 healthy subjects, 14 weeks old mice, and ventricular cardiomyocyte H9c2 were included. The expressions of plasma microRNA-375 in patients with STEMI and NSTEMI and AMI mouse models were remarkably decreased than in controls (*P* < 0.001). The areas under the curve (AUC) of plasma microRNA-375 were revealed 0.939 in STEMI and 0.935 in NSTEMI subjects. Moreover, microRNA-375 levels in H/R-exposed cardiac H9c2 cells were evidently downregulated and significantly increased apoptosis rate and caspase-3 activity levels, while overexpression of miR-375 remarkably reduced apoptosis percentage and caspase-3 levels as compared with normal cells. Furthermore, this study also demonstrated that Nemo-like kinase (NLK), NLK mRNA, and protein expression levels were significantly downregulated in H/R-injured H9c2 cells, on the contrary, H9c2 cells transfected with mimic-miR-375 greatly upregulated NLK mRNA and protein expression. Plasma microRNA-375 may serve as an essential clinical biomarker for diagnosis of early-stage AMI. Mimic expression of miR-375 significantly prevented H/R-induced cardiomyocyte injury by decreasing caspase-3 activity through upregulation of the NLK gene, recommended as a new therapeutic option for AMI patient.

## 1. Introduction

Acute myocardial infarction (AMI) or heart attack is the leading cause of sudden cardiac death all over the world. Rapid hospitalization, an early and exact diagnosis with immediate appropriate therapeutic intervention, may significantly reduce cardiac death rate [1]. Though electrocardiogram (ECG) is a very helpful investigation mechanism for the evaluation of acute chest pain, it has limited sensitivity (50–60%) for the diagnosis of AMI patients. Moreover, currently, cardiac-specific troponin T (cTnT) and troponin I (cTnI) are considered the “gold standard” biomarkers for the diagnosis of AMI patients. However, early diagnosis of AMI is difficult due to the delayed release of troponins from injured cardiomyocytes, which usually started to be increased within 4–6 hours and highest concentrations are reached about 12–24 hours after infarction. Furthermore, currently, it was reported that high-sensitivity cardiac troponin is positive early after ischemia but serial testing is required because troponin is also highly positive in patients with end-stage renal failure, heart failure, and chronic stable angina pectoris. Therefore, the exploration of new ideal biomarkers for early diagnosis of AMI remains to be further explored [2, 3]. Moreover, currently, thrombolytic drugs such as a streptokinase, tenecteplase, and human tissue plasminogen activator have been widely used for the reperfusion therapy in acute myocardial infarction patients, but they have several complications and cannot be used in some selected patients. Therefore, discovering a new ideal drug for the management of all categories of AMI patients is required to reduce the mortality rate [4]. Ischemic/reperfusion- (I/R-) induced myocardial cell injury is considered an important cause (40%-50%) of AMI, which is primarily caused by the restoration of blood flow to the occlusion of coronary artery, that decreases the benefits of active reperfusion therapy. Therefore, reducing the I/R-induced cardiomyocyte damage is a significantly important step for the management of AMI patients. However, there is still a lack of standard therapy for the prevention of I/R-induced myocardial cell injury in clinical practice [5, 6].

MicroRNAs are bioactive small, endogenous, noncoding functional RNA sequences that interact with the specific complementary sequences in the 3′ untranslated region (3′-UTR) of protein-coding mRNAs and represses target gene expression through inhibition of protein translation or transcript of mRNA degradation. Several recent studies have shown that miRNAs play a fundamental role in regulating pathophysiological process in various cardiovascular diseases, including acute myocardial infarction. It is well known that some cardiac-specific miRNAs such as microRNA-208b, microRNA-499, microRNA-1, microRNA-133, and microRNA-378 have been shown to be markedly altered in the plasma following acute myocardial infarction both in patients and animal models; these microRNAs have significant diagnostic impact for acute phase AMI [7, 8]. It has been reported that the expression of miR-340 was significantly downregulated in AMI patients, after I/R in AMI mouse models and hypoxia/reoxygenation- (H/R-) induced H9c2 cells. On the contrary, overexpression of miR-340 markedly reduced caspase-3 activity, apoptosis, and ROS levels in H/R-induced H9c2 cells by the regulation of activator 1(Act1) and NF-*κ*B pathway, and miR-340 could be beneficial for the treatment of I/R injury after AMI [9]. The expression of miR-204 levels was remarkably decreased in hypoxia/reoxygenation- (H/R-) induced rat cardiomyocyte H9c2 cells. Besides, transfection of miR-204 mimics significantly decreased LDH level and apoptosis rate in H/R-induced cardiomyocyte H9c2 cells via regulating sirtuin 1- (SIRT1-) mediated autophagy, indicated that overexpression of miR-204 has a protective effect against myocardial I/R injury [10]. The microRNA-449 level was noticeably elevated in hypoxia-reoxygenation-exposed H9c2 cells. Conversely, inhibition of microRNA-449 evidently decreased cardiomyocte apoptosis and necrosis rate and protected H9c2 cellular damage during H/R condition through the Notch-1 pathway [11]. Moreover, recent studies demonstrated that miR-375 and its target genes were closely associated with myocardial infarction, and circulating miR-375 levels were also abnormally expressed in patients with ST-elevation myocardial infarction [12, 13].

However, Nemo-like kinase (NLK) is considered a mitogen-activated, serine-threonine-protein kinase, a Wnt/*β*-catenin signaling inhibitor; NLK protein coding gene significantly downregulated various inflammatory-mediated transcription factors including transforming growth factor beta and nuclear factor-*κ*B. Some studies have shown NLK protein critically regulated T cell-mediated immunological reaction, apoptosis, and inflammation via activation of Wnt/*β*-catenin molecular mechanisms [14, 15]. Moreover, recent studies established that Wnt signaling pathways are not only implicated in cardiac cell development, proliferation, and differentiation but also significantly engaged in the pathological process of myocardial infarction. Furthermore, it has been also reported that NLK protein molecules are strongly linked with cardioselective miR-208b and hypoxia/reoxygenation-injured cardiomyocytes. However, the function of NLK during hypoxic/reoxygenation-injured H9c2 cells and the relation with miR-375 are largely unknown [16, 17].

Therefore, this research investigated plasma microRNA-375 the expression in acute myocardial infarction patients and mouse models of AMI to evaluate its possible role as an early ideal biomarker for diagnosis of AMI patients. Besides, the current study also examined the potential therapeutic effect of miR-375 to prevent H/R-induced H9c2 cell injury.

## 2. Materials and Methods

### 2.1. Human Study Groups

In this study, ninety ST-elevated AMI (STEMI) patients and seventy-five non-ST-elevated AMI (NSTEMI) patients with an average age of 59 years were enrolled from the cardiovascular unit of the First Affiliated Xiangya Hospital of Central South University, China, from July 2014 to June 2016. STEMI and NSTEMI patients were diagnosed based on the ACC/AHA/ESC/WHF practice guidelines [18]. Ninety age- and gender-matched normal subjects who were undergoing routine medical examinations at the same hospital were also recruited. All of the controls had normal physical examination results, electrocardiographic echocardiography and laboratory tests were normal, and there was no evidence of cardiovascular or cerebrovascular disease. This research was accomplished with the World Medical Association (Declaration of Helsinki) of human experiment guidelines and was also accepted by the Xiangya Hospital Ethics Committee of Central South University. Written consent was taken from AMI patients and healthy subjects before the study. Those AMI patients with chest pain of more than 12 hours, treated with thrombolytic therapy, and with severe heart failure were not included in this study. Five milliliter venous blood samples were collected in K2-EDTA tubes from acute myocardial infarction (chest pain duration within 12 hours) patients and healthy subjects (overnight fasting). Blood samples were immediately centrifuged to obtain pure plasma subsequently stored at -80°C for further analysis.

### 2.2. Establishment of the Mouse AMI Models

Three months old C57BL/6 mice were collected from the veterinary institution of Xiangya School of Medicine, and protocols were carried out following the Chinese national animal care guidelines and approved by the Xiangya animal care board of Central South University. 12 mice were equally distributed among three groups: (1) the control groups; (2) the AMI groups, AMI was established with ligation of the left anterior descending (LAD) coronary artery by 5/0 silk suture, as done by others studies [19, 20]; and (3) the sham groups were subjected to the same surgical procedure except for coronary artery ligation. The mice were sacrificed at 12 hours after LAD ligation and blood samples were immediately collected.

### 2.3. Cell Culture and Hypoxic/Reoxygenation (H/R) H9c2 Cardiomyocytes

From the Shanghai Chinese Academy of Medical Sciences, rat-derived ventricular H9c2 cells were collected and cultured in Dulbecco's modified Eagle's medium with added 10 percent fetal bovine serum (FBS) at a density of (2 × 10^4^/cm^2^) in 6-well plates with maintaining incubator conditions at 37°C, 5% CO2, and 95% air. The hypoxia H9c2 group was established by using serum-free DMEM with no glucose and sodium pyruvate through a modular incubator with 95% N_2_, 5% CO_2_, and 1% O_2_ at 37°C for 12 hours. Subsequently, the cells were reoxygenated by incubating in 95% air and 5% CO2 at 37°C for 6 hours with DMEM containing 10% FBS to mimic ischemic-induced cardiomyocyte injury.

### 2.4. Apoptosis Assay by Hoechst Staining

The Hoechst staining procedure was carried out according to the manufacturer's instruction (Beyotime, Shanghai, China). After 12 hours treatment, firstly, H9c2 cells were cleaned three times by the phosphate-buffered solution subsequently added 2% formalin for 5 minutes then dried in air. Hoechst 33258, a bisbenzimide cell-permeant dye (1 gm/mL) was used for 15 min, at room temperature, under dark conditions to stain the nuclei of the fixed cells. The apoptosis cell number was calculated with compared total number of cells.

### 2.5. Measurement of Caspase-3 Activity Level

Myocardial caspase-3 protein expression was determined with Beyotime caspase-3 activity measuring kits. H/R-treated and normal H9c2 cells (96-well microplates) were properly dissolved by lysis buffer solution, subsequently centrifuged for 10 minutes. Each microplate absorbance was evaluated through a microplate absorbance SpectraMax reader at the wavelength of 405 nm.

### 2.6. Transfections of H9c2 Cells with Mimic-miR-375

Transfections of H9c2 cells were carried out by the Lipofectamine 2000 agent following the manufacturer's protocols (Invitrogen, USA). For overexpression of miR-375, mimic-miR-375 (RiboBio, Guangzhou, China) a kind transfected with 50 nmol/L mimic, and 50 nmol/L mimic-negative control (mimic-NC), which was similar to mimic but with a scramble seeding sequence, served as control.

### 2.7. Luciferase Reporter Gene Analysis

MicroRNA-375 target gene was confirmed through luciferase reporter gene assay tools. Target scan (http://www.targetscan.org/) was used to predict the potential target site for miR-375. Briefly, the 3′ untranslated region (3′-UTR) of NLK and miR-375 were transfected to H9c2 cells with Lipofectamine 2000 reagent. Moreover, 18 hours after transfection of H9c2cells, luciferase activities were determined by dual*-*luciferase reporter assay kit. (Beyotime, Shanghai, China). The data were measured as the ratio of firefly fluorescent activity to the Renilla fluorescence activity.

### 2.8. RNA Isolation and Quantitative Real-Time Polymerase Chain Reaction (qRT-PCR)

From human plasma, mouse plasma, and H9c2 cells, total pure RNA were obtained with the TRIzol reagent (Invitrogen, CA, USA) through the manufacturer's instructions. MicroRNA-375 primer was designed by RiboBio (Guangzhou, China) and the primers of NLK and *β*-actin were designed by Sangon Biotech (Shanghai, China). Moreover, miR-375 and mRNA expression of NLK were examined by using Takara PCR SYBR Green Master Mix Kits with a real-time qRT-PCR system. Synthetic microRNA-156a was considered as an inner control for microRNA-375. RNA isolation and qRT-PCR procedures were briefly described in our previous studies [21].

### 2.9. Western Blot

H9c2 cells were prepared and lysed with cell lysis buffer solution and centrifuged at 12,000 g for 15 min at 4°C. Protein was extracted from whole-cell lysates, and their concentration was measured with BCA protein analyzing kit. Firstly, 30 *μ*g protein samples were separated by 10% SDS-PAGE gel, subsequently; secondly, protein samples were shifted to polyvinylidene difluoride western blotting membranes (PVDF, Roche). Thirdly, PVDF membranes were probed with primary antibodies against NLK (1 : 1000; Santa Cruz), or *β*-actin (1 : 2,000) at 4°C overnight, afterward incubation with horseradish peroxidase-conjugated secondary antibodies (Goat anti-rabbit IgG, 1 : 1000; anti-Mouse IgG, 1 : 6000) at 25°C for 60 min.

### 2.10. Statistics and Data Analysis

Version 21 of Statistical Package for the Social Sciences software (SPSS) was used for the data analysis. Student's *t*-test, the Mann–Whitney test, and one-way ANOVA were used between groups for continuous variation; Fischer's exact or the Chi-square (𝜒2) test were applied among groups for categorical variation. To evaluate the diagnostic impact of plasma microRNA-375 for AMI patients, the area under the curve (AUC) was assessed through a receiver operating characteristic (ROC) curve analysis. All the tests were two-tailed and differences with *P* < 0.05 were considered statistically significant.

## 3. Results

### 3.1. Clinical Parameters of the Study Subjects

In the present study, we included 90 patients with STEMI (median age, 59.17 ± 10.0 years) and 75 patients with NSTEMI (median age, 59.4 ± 10.13 yrs) with well-matched age and gender 90 healthy subjects (median age, 58.2 ± 10.4 yrs). Basic, clinical, laboratory, and drugs information was presented in Table 1. High-sensitivity C-reactive protein, LDH, and CK-MB levels among STEMI and NSTEMI were statistically significant (*P* < 0.001). Body mass index, glucose, high-sensitivity C-reactive protein, LDH, and CK-MB levels were significantly elevated in STEMI and NSTEMI patients than in controls (*P* < 0.001).

### 3.2. Expression Levels of Circulating miR-375 in STEMI and NSTEMI Patients and Healthy Subjects

**Figure 1:**
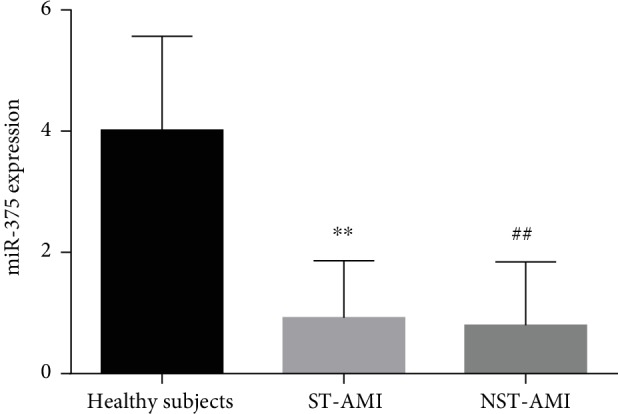
Plasma levels of miR-375 in STEMI and NSTEMI patients and healthy subjects. ^∗∗^*P* < 0.001 healthy subjects versus STEMI, ^##^*P* < 0.001 healthy subjects versus NSTEMI.

The present study demonstrated (*n* = 215) that the relative expression of plasma microRNA-375 levels were remarkably lower in acute STEMI and NSTEMI patients than in healthy subjects (*P* < 0.001). Moreover, expressions of plasma microRNA-375 were downregulated 4.3 times in acute STEMI and 5.04 times in acute NSTEMI patients relative to healthy controls. Besides, plasma levels of microRNA-375 among STEMI and NSTEMI patients were not statistically significant (Figure 1).

### 3.3. Expression Levels of Circulating miR-375 in Mouse AMI Models

**Figure 2:**
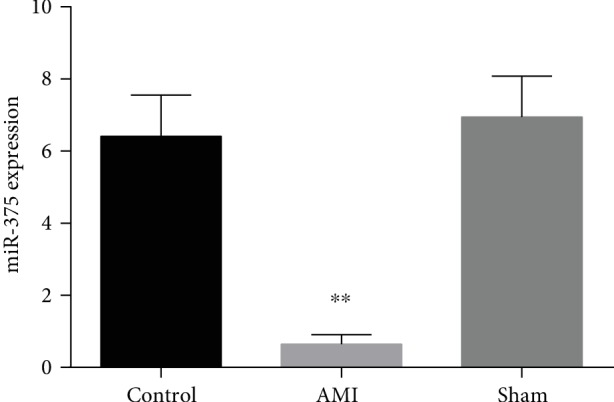
Plasma levels of miR-375 in control, sham and mouse AMI model. ^∗∗^*P* < 0.001 control versus AMI.

The plasma microRNA-375 expression was analyzed from mouse AMI models, sham groups, and controls and revealed that circulatory microRNA-375 expressions were evidently downregulated in mouse AMI than in controls (*P* < 0.001). Circulatory microRNA-375 expressions among sham and controls groups were not statistically significant. Moreover, plasma miR-375 levels were decreased by 10-fold in mice AMI groups as compared with controls (Figure 2).

### 3.4. Diagnostic Impact of Circulating miR-375 in AMI Patients

**Figure 3:**
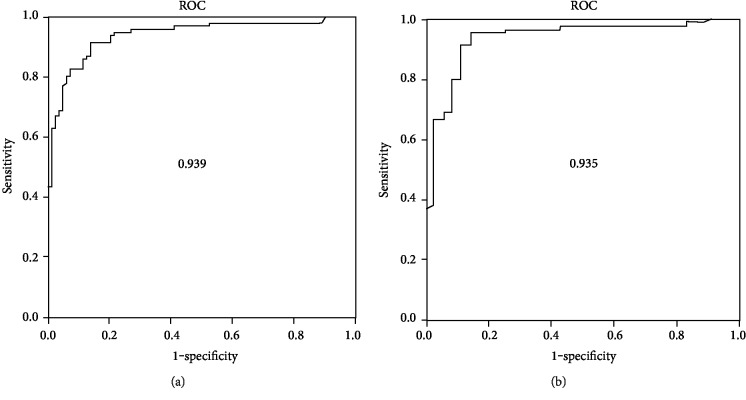
Diagnostic accuracy of plasma miR-375 for AMI patients were constructed by receiver operating characteristic (ROC) curves. (a) Healthy subjects vs STEMI with an AUC of 0.939. (b) Healthy subjects vs NSTEAMI with an AUC of 0.935.

The diagnostic impact of plasma miR-375 as a potential novel biomarker for AMI patient was evaluated with receiver operating characteristic (ROC) curve analysis. As shown in Figure 3, ROC curve analysis of plasma miR-375 exhibited strong separation power between STEMI patients and healthy subjects with an area under curve (AUC) of 0.939, NSTEMI patients and healthy subjects with AUC of 0.935 (*P* < 0.001).

### 3.5. MicroRNA-375 Expression in H9c2 Cells and Luciferase Activity

**Figure 4:**
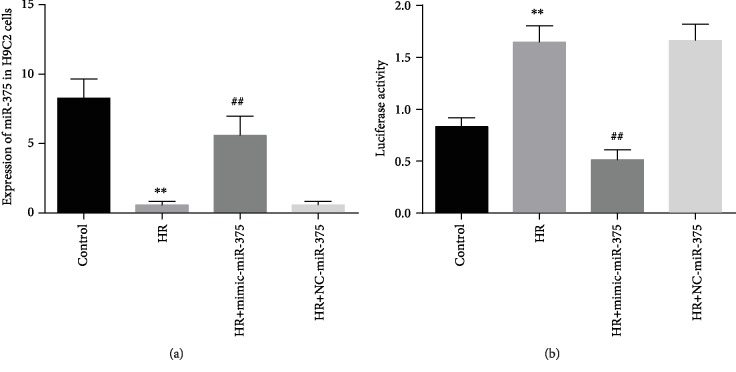
Expression of miR-375 in H9c2 cells and detect target gene. (a) Expression of miR-375 in H9c2 cells. ^∗∗^*P* < 0.001 control versus H/R group, ^##^*P* < 0.001 control versus H/R with mimic-miR-375. (b) Luciferase reporter gene analysis. ^∗∗^*P* < 0.001 control versus H/R, ^##^*P* < 0.01 control versus H/R with mimic-miR-375.

Eighteen hours H9c2 cells were incubated in hypoxic-reoxygenation (H/R) conditions (Figure 4(a)). The expressions of miR-375 were markedly reduced in H/R-exposed H9c2 cells by 14.9-fold than normal H9c2 cells (*P* < 0.001). On the contrary, miR-375 levels obviously upregulated in H/R cells treated with mimic-miR-375 but there was no significant effect on negative control groups. The present study also found that luciferase activity was prominently upregulated in H/R-exposed H9c2 cells. The mimic-microRNA-375 treatment obviously regulated luciferase activity in H9c2 cells during H/R condition (Figure 4(b)).

### 3.6. Role of Mimic-miR-375 on H9c2 Cell Survival in H/R Conditions

**Figure 5:**
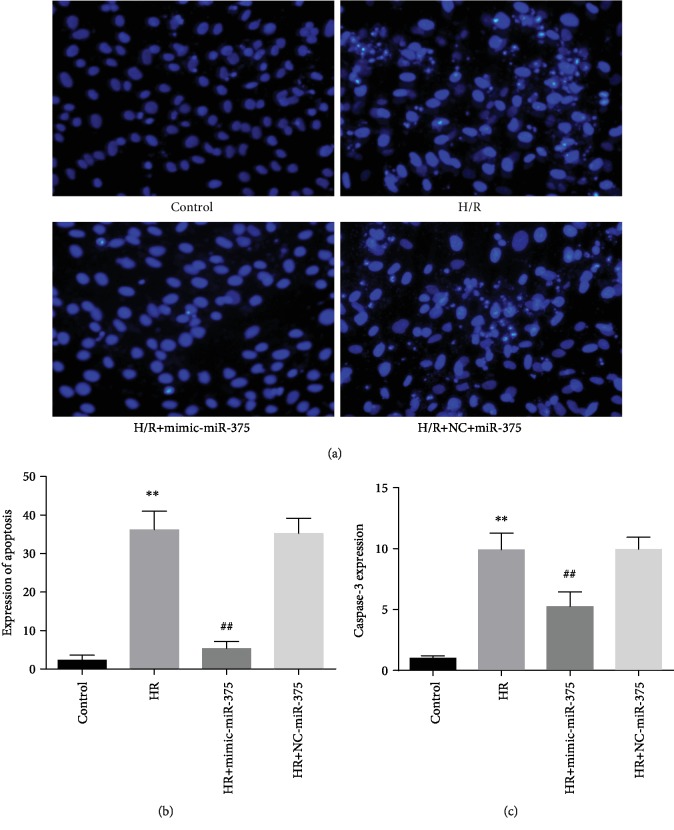
Effect of mimic-miR-375 on H9c2 cell survival in H/R conditions. (a) Hoechst staining (control and H/R ^∗∗^*P* < 0.001; control and mimic-miR-375, ^##^*P* < 0.001; and H/R with negative control (NC)). (b) Expression of apoptosis ratio (control and H/R, ^∗∗^*P* < 0.001; control and mimic-miR-375, ^##^*P* < 0.001; and H/R+NC model). (c) Expression of caspase-3 protein level (control versus H/R, ^∗∗^*P* < 0.001; control with mimic-miR-375, ^##^*P* < 0.001; and H/R+NC model).

Cellular apoptosis rate was detected by Hoechst staining image and noticed that cellular apoptosis rate was markedly elevated in the H/R group as compared with the control; while H/R-induced H9c2 cells were treated with mimic-miR-375, cellular apoptosis rate was tremendously reduced in the H/R group as compared with normal (*P* < 0.001) (Figures 5(a) and 5(b).

Moreover, to examine the mechanism of mimic-miR-375's protective effect on H/R-induced apoptosis in H9c2 cells, the present study analyzed caspase-3 expression levels, which play important roles in regulating the intrinsic pathway of apoptosis in myocardial cells. The current study demonstrated that caspase-3 activity levels were highly expressed in hypoxia-reoxygenation H9c2 models than in controls. Interestingly, for H/R cells treated with mimic-miR-375, caspase-3 expression levels were remarkably decreased as compared with in controls (*P* < 0.001) (Figure 5(c)).

### 3.7. Role of MicroRNA-375 on NLK Expression in H9c2 Cells

**Figure 6:**
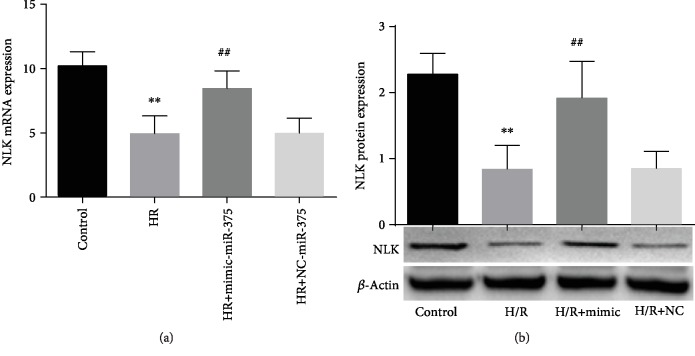
Role of microRNA-375 on NLK expression in H9c2 cells. (a) Expression of NLK mRNA level (control and H/R, ^∗∗^*P* < 0.001; control and mimic-microRNA-375 ^##^*P* < 0.001; and HR + negative control (NC)). (b) Expression of NLK protein level (control and H/R, ^∗∗^*P* < 0.001; control and mimic-microRNA-375, ^##^*P* < 0.001; H/R+NC).

As discussed above, the expression of miR-375 levels was noticeably lower in H/R-exposed H9c2 cells, but after transfection with 50 nmol/L mimic-miR-375 for 18 hours, miR-375 expressions were highly elevated in H/R-induced H9c2 cells relative to controls (*P* < 0.001). Moreover, the present study also found that NLK mRNA and protein expression levels were remarkably decreased in H/R-induced H9c2 cells as compared with normoxic cells. On the contrary, H/R-induced H9c2 cells transfected with mimic-miR-375 significantly upregulated NLK mRNA and protein expression (*P* < 0.001) (Figures 6(a) and 6(b)). These results indicated that mimic-miR-375 outstandingly regulated its target protein and mRNA, which play an essential role to prevent cardiac cell injury in H/R conditions.

## 4. Discussion

The current study investigated the diagnostic and possible therapeutic impact of circulatory microRNA-375 in the early-stage myocardial infarction (AMI) patient. In human, a study demonstrated that circulatory microRNA-35 concentrations were noticeably downregulated and even undetectable in ST-segment elevation MI (STEMI) and non-ST-elevation MI (NSTEMI) than in healthy controls. The diagnostic significance of plasma microRNA-375 as a potential biomarker for AMI patients was explored with ROC curve analysis. Plasma microRNA-375 ROC curves revealed a strong discrimination among STEMI (AUC = 0.939) and NSTEMI (AUC = 0.935) with healthy subjects. This study results are partially supported by a very recent published AMI patients study [22]. In the AMI mouse model, present study found that the plasma levels of miR-375 were significantly decreased after AMI as compared with the control suggesting that cardiac-specific miR-375 could be released into the circulating blood when heart injury occurred. Compared with the result in the AMI patients, current study reported that the plasma levels of miR-375 in AMI mouse models were much higher than those in NSTEMI and STEMI patients. Moreover, present study is also supported by an AMI animal model study [23].

This study selected miR-375 for cell experiment studies because in the present study and also recently several other studies confirmed that microRNA-375 was significantly downregulated in both AMI patients and animal AMI models [23, 24]. In H9c2 cell experiments, cultured H9c2 cardiomyocytes were subjected to 12 hours hypoxia followed by 6 hours reoxygenation and noticed that microRNA-375 remarkably reduced in H/R-exposed H9c2 than normoxic cell. However, Ke et al. reported circulatory miR-375 significantly upregulated in AMI patients while Eryılmaz et al. demonstrated that miR-375 has no significant change in peripheral blood of AMI patients [12, 13]. Accumulating recent evidences reported that microRNA-449, microRNA-423, microRNA-204, and microRNA-665 concentrations were critically altered during H/R-induced cardiomyocyte cell injury. However, H/R-induced H9c2 cells treated with specific inhibition or mimic-miRNAs significantly regulated various cellular inflammatory pathways through their target genes (notch-1, RAP2C, SIRT1, AK1, and Cnr2, respectively) and dramatically reduced oxidative stress, caspase-3 activity, apoptosis, and amazingly protects cardiomyocyte cell injury with obviously increased cellular viability [10, 11, 25, 26]. Very recently, Liang et al. research group demonstrated that microRNA-181c-5p expressions were significantly upregulated in hypoxic reoxygentaion-exposed H9c2 cardiomyocytes and rat myocardial ischemia-reperfusion injury (I-RI). Overexpression of microRNA-181c-5p by mimic microRNA-181c-5p remarkably aggravated cardiomyocyte injury and significantly elevated lactate dehydrogenase and apoptosis rate and markedly decreased cellular viability. On the contrary, inhibition of microRNA-181c-5p by anti-microRNA-181c-5p outstandingly reduced H9c2 cardiomyocyte apoptosis and death rate and evidently increased cardiomyocyte survivability through protein tyrosine phosphatase nonreceptor type 4 (PTPN4) [27].

However, the molecular pathways associated with microRNA-375 in hypoxia/reoxygenation (H/R) exposed cardiomyocyte injury are largely unknown. To explore the underlying molecular mechanism by which miR-375 levels were significantly decreased in H/R-induced H9c2 cells, the present study performed a bioinformatic analysis by using TargetScan software to identify the possible target gene of miR-375 and recognized NLK has a major binding site on 3′ untranslated region (3′-UTR) for microRNA-375.

Nemo-like kinase (NLK) is a protein-coding gene that regulates a number of transcription factors with key roles in cell fate determination. Moreover, a recent study reported that NLK signaling pathway especially plays an important role in cardiac cell development and proliferation. It has been reported that overexpression of miR-208a significantly increased Ang II-induced apoptosis of H9c2 cells via negative regulation of NLK expression, while inhibition of miR-208a noticeably reduced cardiomyocyte apoptosis and cell death through upregulation of NLK [17, 28, 29].

However, previously, miR-375 was considered an important cancer-linked microRNA, and it was widely expressed in tumor and pancreatic tissues. The expression of miR-375 was significantly downregulated in human hepatocellularcarcinoma tissues whereas, upregulation of miR-375 noticeably inhibited human liver cancer cell growth by regulating cell proliferation and apoptosis through its target gene the receptor tyrosine-protein kinase erbB-2 (ErbB2) [30]. Frank et al. demonstrated that miR-375 significantly altered tumor-associated macrophage phenotype and regulated breast cancer cell proliferation and apoptosis via CD36 by targeting TNS3, PXN, and CCL2 expression [31].

A recent study established microRNA-375 abnormally expressed from acute MI patients, AMI rats, and hypoxic-induced cardiomyocytes. Mohsen et al. recognized that miR-375 is a cardiometabolic gene and it has a significant role in coronary artery disease [32]. Expression of miR-375 was significantly changed upon exposure to inflammatory/hypoxic stimulus and also after myocardial infarction. Moreover, interleukin-10 negatively regulated miR-375 signaling pathway and significantly enhanced bone marrow-derived progenitor cell-mediated (BMPAC) myocardial repair and markedly improved LV functions and neovascularization after myocardial infarction in mice [33]. Garikipati et al. demonstrated that miR-375 levels have been abnormally expressed in mouse AMI models and heart failure patients. Interestingly, anti-miR-375 therapy remarkably attenuated inflammatory response, decreased cardiomyocyte death and infarction area, and significantly improved heart function and enhanced angiogenesis through PDK-1/AKT signaling pathways [34].

To the best of our knowledge, this study is the first time demonstrated mimic-miR-375 significantly reduced H/R-induced cardiac cell injury, miraculously reduced cellular apoptosis and caspase-3 activity, and markedly improved cellular viability. Moreover, the present study also found that after transfection H/R-induced H9c2 cells with mimic miR-375 markedly increased both NLK mRNA and NLK protein expression. These results confirmed that NLK is a potential target gene for microRNA-375 and mimic expression of microRNA-375 significantly protect H/R-induced cardiac cell injury by upregulating NLK expression.

However, there are some limitations of the current study needed to be mentioned. First of all, the present study was based on a single institution with comparatively small sample population analysis; this need to be multi-institutional with larger AMI patients' group study to confirm the diagnostic value of this miRNA. Genomic-wide profiling of microRNAs is very important to extract high-quality miRNA expression by using microRNA microarray but in this research microRNA expression was validated by qRT-PCR; microarray technique will be used in the future study [35]. Moreover, the current study only evaluated circulating microRNA-375 expression in AMI patients (chest pain for 12 hours) but did not continue patients' follow-up, as a result correlation between microRNA-375 and severity of the patients' outcome were not discovered. Secondly, present findings were obtained from cultured neonatal rat ventricular-derived H9c2 cells, while human adult ventricular myocytes may not totally have the same working capacity with rat cardiomyocytes. Finally, AMI mouse models need to be treated with mimic miR-375 and we need to also find out the most suitable route for administration and confirm the safeness of mimic-miRNA before applying in a human being.

## 5. Conclusions

Plasma microRNA-375 can be considered a useful clinical diagnostic biomarker for acute MI patients. Upregulation of miR-375 protects apoptosis and cardiac cell injury induced by H/R in H9c2 cells via regulation of the NLK pathway. Present findings indicated that miR-375 may play a preventive role in H/R injury and also provide a new insight for clinical management of AMI patients.

## Figures and Tables

**Table 1:** Clinical parameters of the study subjects.

Clinical parameters	STEMI (*n* = 90)	NSTEMI (*n* = 75)	Healthy subjects (*n* = 90)	*P*_1_	*P*_2_	*P*_3_
Sex (male/female)	53/42	39/36	53/42	1.000	0.645	1.000
Age (years)	59.17 ± 10.0	59.4 ± 10.13	58.2 ± 10.4	0.525	0.456	0.889
Tobacco	73% (65)	72% (54)	58% (52)	0.060	0.073	0.556
Hypertension	78% (70)	76% (57)	0	≠	≠	0.854
Hypercholesterolemia	80% (72)	77% (58)	0	≠	≠	0.706
Diabetes mellitus	32% (29)	31% (23)	0	≠	≠	0.868
Body mass index (kg/m^2^)	24 ± 4.2	25 ± 3.4	22 ± 1.8	0.000	0.000	0.116
Previous MI	11% (10)	6% (5)	0	≠	≠	0.419
Previous CABG	3% (3)	2% (2)	0	≠	≠	1.000
Previous PTCA	6	4	0			0.729
Glucose (mmol/L)	5.94 ± 2.1	6.1 ± 2.1	4.5 ± 0.4	0.000	0.000	0.624
High-sensitivity C-reactive protein (mg/L)	15.17 ± 5.22	13.41 ± 4.66	1.89 ± .1.36	0.000	0.000	0.006
LDH (U/L)	541.211 ± 255.06	512.60 ± 60.83	157.71 ± 22.94	0.000	0.000	0.000
CK-MB (U/L)	594.66 ± 61.56	518.48 ± 93.63	14.51 ± 5.16	0.000	0.000	0.000
cTnT (*μ*g/L)	23.48 ± 6.51	17.4 ± 7.75	0	≠	≠	0.000
Aspirin use	63% (57)	56% (42)	0	≠	≠	0.344
Clopidogrel use	46% (41)	42% (32)	0	≠	≠	0.754
Beta blocker use	84% (76)	84% (63)	0	≠	≠	1.000
Nitrates use	85% (77)	81% (61)	0	≠	≠	0.529
Angiotensin-converting-enzyme inhibitor drugs use	37% (33)	36% (27)	0	≠	≠	1.000
Angiotensin receptor blocker use	14% (13)	12% (8)	0	≠	≠	0.819
Calcium channel blocker use	13% (12)	11% (8)	0	≠	≠	0.640
Lipid-lowering drugs	72%	72%	0	≠	≠	1.000

CABG: coronary artery bypass graft; PTCA: percutaneous transluminal coronary angioplasty; LDH: lactate dehydrogenase; CK-MB: creatine kinase-MB; cTnT: cardiac troponin T. *P*_1_ (controls and STEMI), *P*_2_ (controls and NSTEMI), *P*_3_ (STEMI and NSTEMI).

## Data Availability

The data that have been used in this research are available from the corresponding author upon request.

## References

[1] Benjamin E. J., Muntner P., Alonso A. (2019). Heart disease and stroke Statistics—2019 update: a report from the American Heart Association. *Circulation*.

[2] Han Q. Y., Wang H. X., Liu X. H. (2015). Circulating E3 ligases are novel and sensitive biomarkers for diagnosis of acute myocardial infarction. *Clinical Science*.

[3] Sayed A. S., Xia K., Salma U., Yang T., Peng J. (2014). Diagnosis, prognosis and therapeutic role of circulating miRNAs in cardiovascular diseases. *Heart, Lung & Circulation*.

[4] Bawaskar H. S., Bawaskar P. H., Bawaskar P. H. (2019). Preintensive care: thrombolytic (streptokinase or tenecteplase) in ST elevated acute myocardial infarction at peripheral hospital. *Journal of Family Medicine and Primary Care*.

[5] Neri M., Riezzo I., Pascale N., Pomara C., Turillazzi E. (2017). Ischemia/reperfusion injury following acute myocardial infarction: a critical issue for clinicians and forensic pathologists. *Mediators of Inflammation*.

[6] Sheng Z., Lu W., Zuo Z. (2019). MicroRNA-7b attenuates ischemia/reperfusion-induced H9C2 cardiomyocyte apoptosis via the hypoxia inducible factor-1/p-p38 pathway. *Journal of Cellular Biochemistry*.

[7] Cheng M., Yang J., Zhao X. (2019). Circulating myocardial microRNAs from infarcted hearts are carried in exosomes and mobilise bone marrow progenitor cells. *Nature Communications*.

[8] Sheikh S. A., Xia K., Li F. (2016). GW27-e0556 Clinical impact of circulating miR-208b, miR-499 and miR-378 in acute myocardial infarction. *Journal of the American College of Cardiology*.

[9] Li D., Zhou J., Yang B., Yu Y. (2019). microRNA-340-5p inhibits hypoxia/reoxygenation-induced apoptosis and oxidative stress in cardiomyocytes by regulating the Act1/NF-*κ*B pathway. *Journal of Cellular Biochemistry*.

[10] Qiu R., Li W., Liu Y. (2018). MicroRNA-204 protects H9C2 cells against hypoxia/reoxygenation-induced injury through regulating SIRT1-mediated autophagy. *Biomedicine & Pharmacotherapy*.

[11] Chng J., Wu Q., Lv R. (2018). MicroRNA-449a inhibition protects H9C2 cells against hypoxia/reoxygenation-induced injury by targeting the Notch-1 signaling pathway. *Cellular Physiology and Biochemistry*.

[12] Wu K., Zhao Q., Li Z. (2018). Bioinformatic screening for key miRNAs and genes associated with myocardial infarction. *FEBS Open Bio*.

[13] Eryılmaz U., Akgüllü Ç., Beşer N., Yıldız Ö., Kurt Ömürlü İ., Bozdoğan B. (2016). Circulating microRNAs in patients with ST-elevation myocardial infarction. *Anatolian Journal of Cardiology*.

[14] Zhang H. H., Li S. Z., Zhang Z. Y. (2014). Nemo-like kinase is critical for p53 stabilization and function in response to DNA damage. *Cell Death and Differentiation*.

[15] Fleskens V., Minutti C. M., Wu X. (2019). Nemo-like kinase drives Foxp3 stability and is critical for maintenance of immune tolerance by regulatory T cells. *Cell Reports*.

[16] Yan X., Liu J., Wu H. (2016). Impact of miR-208 and its target gene Nemo-like kinase on the protective effect of ginsenoside Rb1 in hypoxia/ischemia injuried cardiomyocytes. *Cellular Physiology and Biochemistry*.

[17] Liu R., Khalil H., Lin S. J., Sargent M. A., York A. J., Molkentin J. D. (2016). Nemo-like kinase(NLK) is a pathological signaling effector in the mouse heart. *PLoS One*.

[18] Thygesen K., Alpert J. S., Jaffe A. S. (2012). Third universal definition of myocardial infarction. *Circulation*.

[19] Xu L., Yates C. C., Lockyer P. (2014). MMI-0100 inhibits cardiac fibrosis in myocardial infarction by direct actions on cardiomyocytes and fibroblasts via MK2 inhibition. *Journal of Molecular and Cellular Cardiology*.

[20] Maejima Y., Kyoi S., Zhai P. (2013). Mst1 inhibits autophagy by promoting the interaction between Beclin1 and Bcl-2. *Nature Medicine*.

[21] Ali Sheikh M. S., Salma U., Zhang B., Chen J., Zhuang J., Ping Z. (2016). Diagnostic, prognostic, and therapeutic value of circulating miRNAs in heart failure patients associated with oxidative stress. *Oxidative Medicine and Cellular Longevity*.

[22] Wang Y., Chang W., Zhang Y. (2019). Circulating miR-22-5p and miR-122-5p are promising novel biomarkers for diagnosis of acute myocardial infarction. *Journal of Cellular Physiology*.

[23] Wang G. K., Zhu J. Q., Zhang J. T. (2010). Circulating microRNA: a novel potential biomarker for early diagnosis of acute myocardial infarction in humans. *European Heart Journal*.

[24] Baulina N., Osmak G., Kiselev I. (2018). NGS-identified circulating miR-375 as a potential regulating component of myocardial infarction associated network. *Journal of Molecular and Cellular Cardiology*.

[25] Luo H., Li X., Li T. (2019). microRNA-423-3p exosomes derived from cardiac fibroblasts mediates the cardioprotective effects of ischaemic post-conditioning. *Cardiovascular Research*.

[26] Yu J., Yang W., Wang W. (2019). Involvement of miR-665 in protection effect of dexmedetomidine against Oxidative Stress Injury in myocardial cells via CB2 and CK1. *Biomedicine & Pharmacotherapy*.

[27] Liang G., Cai Y., Ying F. (2019). miR-181c-5p exacerbates hypoxia/reoxygenation-induced cardiomyocyte apoptosis via targeting PTPN4. *Oxidative Medicine and Cellular Longevity*.

[28] Palevski D., Levin-Kotler L. P., Kain D. (2017). Loss of macrophage Wnt secretion improves remodeling and function after myocardial infarction in mice. *Journal of the American Heart Association*.

[29] Huang Y., Yang Y., He Y., Hunag C., Meng X., Li J. (2016). MicroRNA-208a potentiates angiotensin II-triggered cardiac myoblasts apoptosis via inhibiting Nemo-like kinase (NLK). *Current Pharmaceutical Design*.

[30] Li L., Jia L., Ding Y. (2018). Upregulation of miR-375 inhibits human liver cancer cell growth by modulating cell proliferation and apoptosis via targeting ErbB2. *Oncology Letters*.

[31] Frank A. C., Ebersberger S., Fink A. F. (2019). Apoptotic tumor cell-derived microRNA-375 uses CD36 to alter the tumor- associated macrophage phenotype. *Nature Communications*.

[32] Ghanbari M., Franco O. H., de Looper H. W., Hofman A., Erkeland S. J., Dehghan A. (2015). Genetic variations in microRNA-binding sites affect microRNA-mediated regulation of several genes associated with cardio-metabolic phenotypes. *Circulation: Cardiovascular Genetics*.

[33] Garikipati V. N., Krishnamurthy P., Verma S. K. (2015). Negative regulation of miR-375 by interleukin-10 enhances bone marrow-derived progenitor cell-mediated myocardial repair and function after myocardial infarction. *Stem Cells*.

[34] Garikipati V. N. S., Verma S. K., Jolardarashi D. (2017). Therapeutic inhibition of miR-375 attenuates post-myocardial infarction inflammatory response and left ventricular dysfunction via PDK-1-AKT signalling axis. *Cardiovascular Research*.

[35] Chen Z., Hu Z., Lu Z. (2013). Differential microRNA profiling in a cellular hypoxia reoxygenation model upon posthypoxic propofol treatment reveals alterations in autophagy signaling network. *Oxidative Medicine and Cellular Longevity*.

